# Challenges and future prospects of antibiotic therapy: from peptides to phages utilization

**DOI:** 10.3389/fphar.2014.00105

**Published:** 2014-05-13

**Authors:** Santi M. Mandal, Anupam Roy, Ananta K. Ghosh, Tapas K. Hazra, Amit Basak, Octavio L. Franco

**Affiliations:** ^1^Central Research Facility, Department of Chemistry and Department of Biotechnology, Indian Institute of Technology KharagpurKharagpur, India; ^2^Division of Pulmonary and Critical Care Medicine, Department of Internal Medicine, University of Texas Medical Branch at GalvestonGalveston, TX, USA; ^3^Centro de Análises Proteômicas e Bioquímicas, Pós-Graduação em Ciências Genômicas e Biotecnologia, Universidade Católica de BrasíliaBrasilia, Brazil

**Keywords:** antibiotics, multi-drug-resistant pathogens, infection control

## Abstract

Bacterial infections are raising serious concern across the globe. The effectiveness of conventional antibiotics is decreasing due to global emergence of multi-drug-resistant (MDR) bacterial pathogens. This process seems to be primarily caused by an indiscriminate and inappropriate use of antibiotics in non-infected patients and in the food industry. New classes of antibiotics with different actions against MDR pathogens need to be developed urgently. In this context, this review focuses on several ways and future directions to search for the next generation of safe and effective antibiotics compounds including antimicrobial peptides, phage therapy, phytochemicals, metalloantibiotics, lipopolysaccharide, and efflux pump inhibitors to control the infections caused by MDR pathogens.

## INTRODUCTION

Antibiotics are essential therapeutics, commonly used to control bacterial infections. They are one of the most significant contributions to modern science and have proved to be of vital importance in the dramatic rise in average life expectancy. Nevertheless, antimicrobial resistance is clearly ready to jeopardize this development now and in the near future. Four years after the successful introduction of penicillin, the first appearance of an antibiotic-resistant strain was reported during World War II (Levy, 2002). Maurois (1959) warned about the deadly fact of antibiotic resistance, stating that the inappropriate use of penicillin could lead to the selection of resistant “mutant forms” of *Staphylococcus aureus* causing serious infections in the host. Since then, acquired bacterial resistance has caused nosocomial infections with morbidity and mortality in hospitalized patients, and to general alarm these infections have been observed spreading to immune depressed patients (Michael et al., 2013). Each year in the United States of America, at least two million persons become infected with antibiotic-resistant bacteria and at least 23,000 people die every year as a direct result of such infections (Antibiotic resistance threats in the United States, 2013). Many examples of resistant strains could be cited. Between 1987 and 2004, high levels of penicillin resistance in *Streptococcus pneumoniae* were observed, reaching almost 20%. At the same time, a 50% increase in methicillin-resistant in *Staphylococcus aureus* (MRSA) was also observed (Herrmann and Laxminarayan, 2010). Additionally, very frequent and inappropriate use of antibiotics, lack of educational awareness and regulatory authority regarding antibiotic usage, production, and marketing as well the lack of infection control in hospitals and inadequate water and sanitation in the community makes the situation worse. Spread of Gram-negative bacilli resistance is an emerging problem of Asian countries.

Surveillance study on the resistance on *Salmonella enterica serotype Typhi (Salmonella typhi) and Paratyphi (Salmonella paratyphi)* conducted in seven Asian countries (Korea, Taiwan, Vietnam, Philippines, Singapore, Hong Kong, and Sri Lanka) from 2002 to 2004 emerged high rates of resistance against normally used antibiotics. In Vietnam, the proportion of multi-drug-resistant (MDR) strains was 30% higher than in the other six countries (Chuang et al., 2008).

Currently, application of antibiotics seems to be the main anti-infective solution for patients in major trauma or in intensive care. Furthermore, similar antibiotic therapies are generally applied to prevent post-surgery infections or in the treatment of life-threatening infection in patients with various kinds of cancer. These treatments, however, have become more difficult due to pathogen resistance. Antibiotic resistance has led a string of researchers to work on alternative strategies to “reset the clock” for resistance levels in particular pathogens. Although some promising antibiotics have reached phase three trials, and many of them are under phase two, the continuous development of new compounds is extremely important, as will be described below. In this context, this review article sheds some light on future directions to search for the next generation of antimicrobial compounds and examines strategies like antimicrobial peptides (AMPs), phage therapy, phytochemicals, metallo-antibiotics, lipopolysaccharide (LPS) inhibitors, and efflux pump inhibitors to control the infections caused by MDR bacterial pathogens (Table 1).

**Table 1:** Major types of antimicrobial compounds with their mechanisms of action.

Future therapy	Mechanism	Contemporary strategies to improve activity
Antimicrobial peptides	Attach and insert into membrane bilayers to form pores by “barrel-stave,” “carpet,” or “toroidal-pore” mechanisms. DNA and macromolecule synthesis inhibitors.	Optimization of peptide length and content of their sequences.
		Conversion into peptidomimetics.
		Generation of targeted antimicrobial peptides (Peptide antibiotic conjugation).
		Generation of antimicrobial peptides as prodrug candidates.
		Antimicrobial peptides loaded into nanoparticle or micelles for sustained release.
Phage therapy	Bacteriophages are viruses that act as pathogens against bacteria and completely lyse the bacteria.	Genetically engineered phages.
		Genetically engineered phase as antibiotic delivery.
		Engineered bacteriophage for phage targeted drug delivery.
		Scale up of endolysin production.
Phytochemicals	Multiple actions.	Search for novel compounds and cost-effective methods of extraction and purification of phytochemical.
		Transgenic production in plant and microbial system to enhance number of novel compounds.
		Search for endophytic fungal metabolomics for the production of novel compound of host.
		Synthesis and modification of natural structure and analogs.
Metalloantibiotic	Increased spectrum of conventional antibiotic action.	Synthetic or semi-synthetic antimicrobial compound development attaching metal to its structure.
		*In situ* reducing and capping of metal nanoparticle with enhanced antimicrobial activity.
Efflux pump inhibitor	Molecules to inhibit the active protein pump in the bacterial cell.	Chemical synthesis of effective efflux pumps inhibitor.
		Screening of efflux pump inhibitors from natural origin and modifying this compound synthetically.
		Rationally designed transmembrane peptide mimics.

## OVERVIEW OF MECHANISMS OF ANTIBIOTIC RESISTANCE

Antibiotic resistances are commonly related to bacterial mutations. Such mutations could occur due to the selection pressure exerted by the random and inappropriate use of bactericidal or bacteriostatic agents. Under continued selection pressure, the selected bacteria may become resistant to antibiotics and spread to other bacteria by transferring the resistance gene (Levy and Marshall, 2004). These unique resistance capabilities are generally subdivided into four major issues. First is enzymatic drug inactivation, as observed in the case of beta-lactamases (Davies, 1994). Second, resistance could be related to alteration of specific target sites (Spratt, 1994), as observed in the case of penicillin-binding proteins (PBPs) in MRSA. Third, bacteria may acquire several genes for a metabolic pathway. This alters bacterial cell walls and thus makes antimicrobial agents incapable of binding to a bacterial target. Finally, the fourth issue is the reduction in drugs’ cellular uptake (Smith, 2004). In this case, para-amino benzoic acid (PABA) is an important precursor for bacterial folic acid and nucleic acid synthesis. Some sulphonamide-resistant bacteria do not require PABA, instead using preformed folic acid as observed in mammalian cells. As a result, a decrease in drug permeability or an increase in active efflux of the drug across the cell surface causes a decrease in drug accumulation in cellular compartments (Nakaido, 1994). Bacteria may also acquire efflux pumps that extrude the antibacterial agent from the cell before it can reach its target site and exert its deleterious effect. This resistance mechanism plays a vital role in reducing the clinical efficacy of antibiotics. Moreover, the overproduction of efflux pumps is generally accompanied by a resistance improvement of two or more structurally unrelated antibiotics and significantly contributes to the emergence of MDR pathogens. There are five major families of efflux transporters, MFS (major facilitator superfamily), MATE (multi-drug and toxic compound extrusion), RND (resistance nodulation cell division) superfamily, SMR (small multi-drug-resistance), and ABC (ATP-binding cassette) transporters (Eda et al., 2011; Spellberg et al., 2013). All these mechanisms of resistance have been targeted by the scientific community finding the search for novel antibiotics with multiple functions, as described above.

## ANTIMICROBIAL PEPTIDES

**FIGURE 1:**
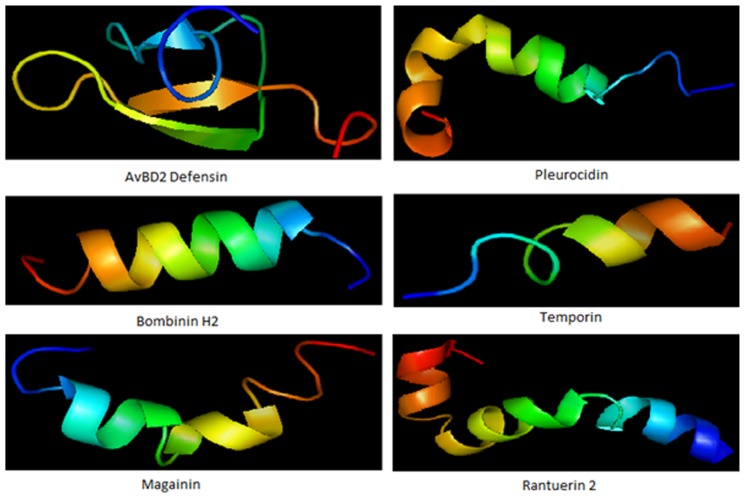
**Some representative structure of antimicrobial peptides**. Structure represents beta defensin peptide from avian (AvBD2); an amphipathic alpha-helical peptide from skin mucous of *Pleuronectes americanus* (Pleurocidin); an antimicrobial hemolytic peptide from skin of *Bombina variegata* (bombinin H2); skin secretion of *Rana temporaria* (temporin); another antimicrobial peptide frog *Xenopus laevis* (magainin) and peptide with helix-turn-helix motif from *Rana cascadae* (ranatuerin).

Over the last few decades, several AMPs have been identified (**Figure 1**) and rigorously investigated as alternatives to antibiotics. They have been widely tested on the antibiotic-resistant bacterial infections (Hancock and Lehrer, 1998; Ganz, 2003). AMPs are the first line of defense in various organisms including plants (Mandal et al., 2013; Roy et al., 2013), humans, insects and other invertebrates, amphibians, birds, fish, and mammals (Martin et al., 1995; Wang and Wang, 2004). Most AMPs are cationic in nature and generally possess a specific amphipathic conformation. These key players in defense systems have attracted extensive research attention worldwide. AMPs are generally short (<100 amino acid residues), positively charged and amphiphilic in nature. This allows them to bind and insert themselves into membrane bilayers to form pores by “barrel-stave,” “carpet,” or “toroidal-pore” mechanisms (Matsuzaki et al., 1996; Oren and Shai, 1998; Yang et al., 2001). Several data previously provided suggest that translocate peptides may alter cytoplasmic membrane septum formation (Salomon and Farias, 1992; Shi et al., 1996), inhibiting cell wall synthesis (Brotz et al., 1998), bind to nucleic acids (Yonezawa et al., 1992; Brotz et al., 1998; Park et al., 1998), inhibit nucleic acid synthesis (Yonezawa et al., 1992; Boman et al., 1993; Silvestro et al., 1997; Subbalakshmi and Sitaram, 1998), impede protein synthesis (Yonezawa et al., 1992; Boman et al., 1993; Silvestro et al., 1997; Subbalakshmi and Sitaram, 1998), or inhibit enzymatic activity (Andreu and Rivas, 1998; Brogden, 2005). These features make some AMPs most acceptable as a novel antibiotic class and they can complement conventional antibiotic therapy (Marshall and Arenas, 2003; Hancock and Sahl, 2006; Jenssen et al., 2006).

The activity of AMPs may vary by switching amino acid composition, amphipathicity, cationic charges and size. In this context, AMPs can be improved through the amalgamation of hydrophobic or charged amino acids, which has been revealed to modify the selectivity for fungal and bacterial membranes (Davies, 1994; Levy and Marshall, 2004). In this approach, different strategies in designing novel peptides have been pursued, as described in several studies (Tamamura et al., 1994; Pini et al., 2005). There are descriptions of potential AMPs from natural sources helping to design “tailor-made” AMPs owing to their easy availability through solid-phase peptide synthesis (SPPS; Martin et al., 1995; Wang and Wang, 2004). Several synthetic analogs of a number of naturally occurring AMPs have been made, in an effort to identify significant structural features that contribute to their enhanced activities *in vitro* against Gram-positive and Gram-negative bacteria, fungi as well as some enveloped viruses (Jenssen et al., 2006).

Cationic peptides contain cationic residues including arginines and lysines. These residues are involved in the attraction of the negatively charged bacterial cell surface. Structure–function studies of host defense α-helical peptides have been carried out, with the aim of designing diverse engineered cationic antimicrobial peptides (eCAPs; Tencza et al., 1999; Jing et al., 2003; Phadke et al., 2003; Deslouches et al., 2005a, b; Chan et al., 2006; Andrushchenko et al., 2007, 2008; Nguyen et al., 2010). Their focus was on structure–function relationships of host-derived synthetic AMPs. A series of eCAPs, called a lytic base unit (LBU) series, formed of only Arg and Val, has been engineered to fold onto a flawless amphipathic helical motifs in the occurrence of lipid membranes or membrane mimitope solvents. Furthermore, Rozek et al. (2000) explored the structure of the bovine AMP indolicidin linked to dodecylphosphocholine and sodium dodecyl sulfate micelles. Haney et al. (2012a) showed how specific amino acid side chains influence the antimicrobial activity and structure of bovine lactoferrampin. All the data have revealed the membrane perturbation properties of Arg and Trp. Further research on the optimization of the amphipathic helix has also been carried out. An unusual series of eCAPs (6–18 residues long) has recently been reported (Deslouches et al., 2013) to have broad and potent *in vitro* activity against MDR pathogens. These eCAPs consist exclusively of Arg on the hydrophilic face and Trp on the hydrophobic face.

Peptide modification, formulation, and delivery technologies have also been explored to overcome the shortcomings of pharmacokinetics, bioavailability, and toxicity (Cole et al., 2003; Devocelle, 2012; Haney et al., 2012b). In this regard, several strategies have been used, such as the optimization of peptide length and content, offering an increase in selective antibacterial activity. Optimization is generally done by minimizing the peptide length or switching the peptide surface properties and systematically substituting each residue. For these cases, computer-assisted AMP design is very useful for an accurate estimation or prediction of the desired biological activity from the primary peptide structure (Fjell et al., 2012). Moreover, conversion into peptidomimetics techniques is able to improve the pharmacokinetic properties of AMPs, since peptidomimetic structures are resistant to proteolysis.

Other strategies have also been applied to discovering novel antimicrobials. Recently classical antibiotics were conjugated for host defense peptide sequence, thereby increasing selectivity and effectiveness against bacteria (Pokrovskaya and Baasov, 2010; Arnusch et al., 2012). Additionally, the generation of AMP pro-drug candidates has also been focused (Hancock, 2001; Stella, 2004; Gordon et al., 2005; Desgranges et al., 2012). Last but not least, AMPs have been nanoencapsulated by strategies including self-assembly, liposomes, polymeric structures, hydrogels, dendritic polymers, nanospheres, nanocapsules, carbon nanotubes, and DNA cages. These strategies offer enhanced antimicrobial activity, a reduction in collateral effects and also a clear protection from metabolic degradation (Urbán et al., 2012; Roy et al., 2013).

**FIGURE 2:**
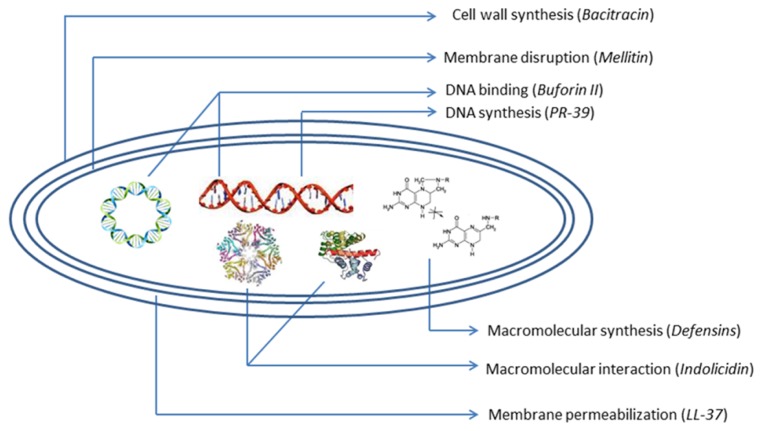
**Schematic diagram of antibacterial peptides with mechanism of action**.

The problem regarding the costs of synthesis and screening, systemic, high manufacturing costs, and local toxicity, susceptibility to proteolysis, sensitization, and allergic responses after repeated uses of cationic membranolytic AMPs is the key barrier in successful clinical application (Gordon et al., 2005). But AMPs, with their unique multidirectional mode of action (**Figure 2**) and broad-spectrum activities, rapid onset of killing, potentially low levels of induced resistance seem to represent one of the most promising future strategies to overcome increasing antibiotic-resistant pathogens. These desirable and remarkable compounds are now being studied extensively, and attempts to create them synthetically are being made by both academics and industries. Preclinical and clinical studies of AMPs are being focused more in order to overcome the problems. Efforts to produce AMPs on an industrial large scale are now also in progress (Giuliani et al., 2007). A good number of synthetic AMPs and at least 15 peptides or mimetics are undergoing advanced clinical trials or have completed trials as antimicrobial or immunomodulatory agents (Fjell et al., 2012). Antimicrobial cationic lipopeptides like polymyxin, gramicidin S, bracitracin, and cationic lantibiotic nisin have offered clinical efficacy and are used widely (Laverty et al., 2010). In the future, the inappropriate use of AMPs may lead to more resistant forms of microorganisms that produce deadly infections. In this context, innovative computer-assisted design strategies can strengthen the ongoing development of next-generation therapeutic peptides and peptide mimetics.

## PHAGE THERAPY

The use of bacteriophages in controlling bacterial infections is also a promising therapeutic option. Bacteriophages are bacterial viruses that act as pathogens against bacteria. They show the ability of specifically attacking and killing only host bacterial cells at the end of infection process (Sulakvelidze et al., 2001). After the first isolation of bacteriophage in 1917, an oral phage preparation to treat bacterial dysentery was used (d’Herelle, 1917). Phages are then extensively used and developed mainly in former Soviet Union countries. Several commercial laboratories and companies in the USA, France and Germany developed phase products (Hausler, 2006). The golden age in use of phage was in the 1930s, but with antibiotics discovery, the progress of phage research and use was reduced.

**FIGURE 3:**
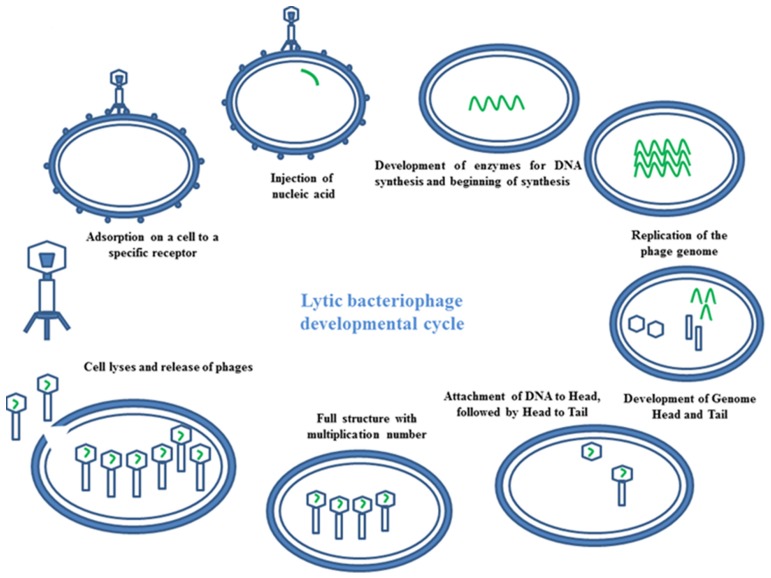
**Mechanism of phage therapy**. Image represents the schematic diagram of developmental cycle of lytic bacteriophage.

Bacteriophages have the ability to interfere between two cycles lysogenic or lytic (Temperateness). In the lytic phage, the viral DNA exists as a separate molecule within the bacterial cell, and replicates separately from the host bacterial DNA. Each phage follows a unique pathway to control bacteria. Some of them show a lytic infection cycle upon infecting their bacterial host. In this case, they grow in high numbers in bacterial cells, leading to cellular lyses. At the end of the cycle, a release of newly formed phage particles is observed (**Figure 3**). Using the lysogenic pathway, the phage genome integrates as part of the host genome. It stays in a dormant state as a prophase for extended periods of time. Adverse environmental conditions for the host bacterium may activate the prophase, turning on the lytic cycle. At the end, the newly formed phage particles are ready to lyse the host cell (Skurnika and Strauch, 2006).

It has been noted that the bacterial mechanism of resistance to phage seems to be lower when compared to antibiotics, which are prone to bacterial resistance. The exponentially higher growth kinetics generally overcomes bacterial growth (López et al., 1997). Moreover, phages seem to show an extra advantage over common antibiotics, which are generally reduced by metabolism and excretion, with several repeated administrations being necessary (Inal, 2003). In the case of phages, increasing titers during different periods removes the need for repeated doses. Additionally, high specificity for a particular bacterium does not disturb the host-organism, so that phages do not affect commensal intestine micro-flora, which is generally a side-effect in the case of antibiotic ingestion (Inal, 2003). Although phages may carry a virulence factor or toxic genes (Mesyanzhinov et al., 2002; Brüssow et al., 2004; McGrath et al., 2004), a full knowledge of phage genome sequences can address the possible complications during phage therapy (Skurnika and Strauch, 2006). Specific and non-toxic phages with high therapeutic index can be applied to reduce the chances of opportunistic pathogens.

Generally whole virulent phages are used as antibacterials. Genetically modified phages are now also being studied, and have been reported as useful in delivering antimicrobial agents to bacteria. Westwater et al. (2003) documented the use of a non-lytic phage to precisely target and deliver DNA encoding bactericidal proteins to target. Engineered bacteriophages can also enhance the killing of antibiotic-resistant bacteria, persistent cells and biofilm cells. They reduce the number of antibiotic-resistant bacteria that ascend from an antibiotic-treated population, and act as a robust adjuvant for other bactericidal antibiotics (Lu and Collins, 2009; Kaur et al., 2012). Moreover, a study on endolysins, which are hydrolytic enzymes secreted by bacteriophage, has revealed potential antimicrobial activity (Nelson et al., 2012).

Treatment using the phage is not approved yet in countries other than Russia and Georgia. Phages are currently being used therapeutically only in the Russia and Georgia to treat extreme bacterial infections where conventional to antibiotics do not respond (Karl, 2004; Parfitt, 2005). The use of phage technology for bacterial control could also be applied in veterinary products focusing on animal health. Bacterial resistance to antibiotics is a serious concern for animal production, which could also be addressed by phage therapy in the near future. Based on several features of bacteriophages, it has even been proposed that they be used in food to prevent bacterial foodborne infections in food products and on food contact surfaces (Borysowski et al., 2011). The example includes LMP-102, which was regarded safe for use as food additive in meat and poultry products as an antibacterial agent against *Listeria monocytogenes* (Daniells, 2006).

Archaebacteria-specific viruses or archeophages are the most recent discoveries to show successful results in controlling bacterial spread. Some bacteriophages have been used as anti-infective agents, such as bacteriophage lysins and bacteriophage tail-like bacteriocins (Schuch et al., 2002; Huff et al., 2004). For example, a G phage lysine, PlyG, can effectively control *Bacillus anthraces* in mice models (Schuch et al., 2002). Phages are recently being commercialized in several areas of biological applications. Companies throughout the world like Intralytix (Baltimore, MD, USA -product based on food safety), Phage Biotech Ltd have (Rehovot, Israel -Anti-Pseudomonas infectives), BioControl (Southampton, UK -*Pseudomonas* infections of the ear), EBI Food Safety (Wageningen, Netherlands -product based on Food Safety. cocktail of phage against *Listeria*), JSC Biochimpharm (Tbilisi, Republic of Georgia -mixture of phage lysates), Gangagen (India -phage against *Staphylococcus aureus*), Omnilytics (Salt Lake City, UT, USA -Agricultural use) etc., (Housby and Mann, 2009). Despite the clear benefits of phage therapy, some problems like development of antibodies after repeated treatment with phages, rapid uptake and inactivation of phages by spleen, contamination of therapeutic phage preparations with endotoxins from bacterial debris, limited host range, regulation, bacterial resistance to phages, engineering, bacterial lysis side effects and delivery should be scrutinized more thoroughly to make these potent therapeutics in the near future (Inal, 2003; Lu and Koeris, 2011). In summary the phage theraphy contribution in terms of continued investments in research, development, and clinical trials from the public and private sectors are needed to overcome regulatory and technical hurdles.

## PHYTOCHEMICALS

Phytochemicals are the tremendous gift of nature. They are the secondary metabolites basically found in plants for specific functional purposes. In most cases, these substances act as a plant defense mechanism against microorganisms, insects and herbivores. At the dawn of civilization, phytochemicals, in the form of plants, were the only weapon in a struggle between man and microbes. In recent times they have stimulated the same interest both as fundamental sources of new chemical diversity and integral components of today’s pharmaceutical compendium. But with ever more incidents of MDR pathogens, the search for new chemicals from plants offering a wide range of activity is the recent focus of researchers.

**FIGURE 4:**
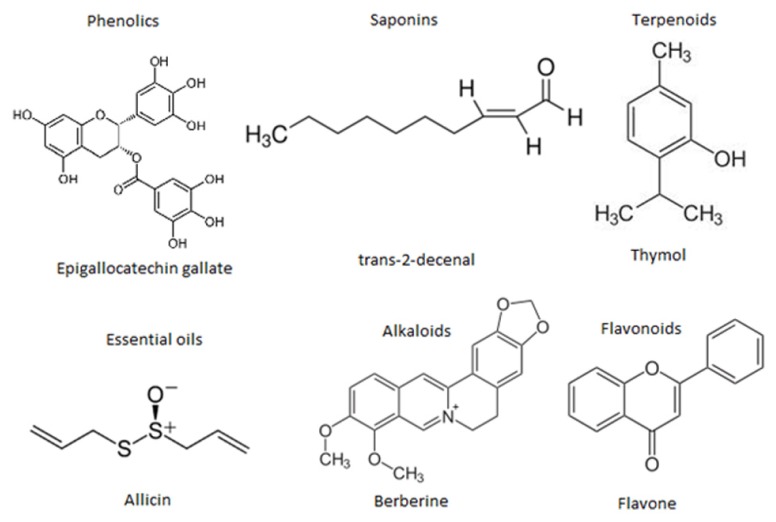
**Representative chemical structures of some antimicrobial phytochemicals**. Phytochemicals like phenolics, saponins, essential oils, terpenoids, alkaloids, and flavonids are the major classes showed antibacterial activity.

At the moment thousands of compounds derived from plants have been listed as antimicrobials. Phytochemicals within the group of phenolics, terpenoids, essential oils, alkaloids, proteins and peptides, possess potent antimicrobial potential with varying mode of action (Cowan, 1999; **Figure 4**). Modern research is not only confined to searching for antimicrobial compounds but has also found several enzymatic inhibitors. Berberine is a hydrophobic cation found in common barberry (*Berberis* species) plants and the medicinal plant goldenseal (*Hydrastis canadensis*). Although the immutable targets and positive charge (facilitating active accumulation in bacterial cells) makes berberine an efficient antibacterial, the fact that it is readily extruded by pathogen-encoded MDR pumps rendered it ineffective. This limitation was overcome by finding and applying another barberry-isolated compound, 5′-methoxyhydnocarpin, which acts by blocking so-called major facilitator MDRs of Gram-positive bacteria. In combination, the two act as potent antimicrobials (Hsieh et al., 1998; Stermitz et al., 2000; Lewis, 2001; Boucher et al., 2009). Furthermore, a newly identified compound, 4-[*N*-(1,8-naphthalimide)]-*n*-butyric acid, showed inhibition activity of the *Vibrio cholerae* transcriptional regulator ToxT. Cholera toxin and the toxin-co-regulated pilus are regulated by ToxT. This compound was tested in an animal model, with infant mice, and was also reported to protect intestinal colonization by *V. cholera* (Hung et al., 2005; Lewis and Ausubel, 2006).

Strategies like study the combo effects of antibiotic and phytochemical are recently been studied. Recently interaction studies are drawn to study the associated effects of antibiotic and phytochemicals (Sakharkar et al., 2009; Jayaraman et al., 2013). A current study revealed that phytochemical-antibiotic conjugates have multitarget inhibitors of *Pseudomononas aeruginosa* GyrB/ParE and DHFR was extremely effective (Jayaraman et al., 2010).

In the search for novel compounds and cost-effective methods of extraction, purification of phytochemicals is a major concern. But the mode of action derived from the structure may lead new potent antibiotic and precursor molecule. For this, genetically modified plant and microbial systems are also now being tried to explore an enhanced number of novel compounds. Additionally, novel antimicrobial structures have been created synthetically. Synthetic analogs and modification of the structure may lead to the discovery of novel structures with a broader spectrum of activity.

## METALLOANTIBIOTICS

Among different strategies to discover novel antibiotics, the incorporation of metal ions in antimicrobial compounds seems to be promising. Metal ions perform an essential role in the functions of synthetic and natural metalloantibiotics, being involved in very specific interactions of such antibiotics with membranes, proteins, nucleic acids, and several other biomolecules. This makes metal ions effective in antibiotic structure or as an operative linkage offering unique and specific bioactivities. Although several antibiotics do not possess any metal ions in their structure, others require metal ions for their proper functional activities. In some cases metal ions are bound tightly to the antibiotic structure and regulate its action (Ming, 2003). Bacitracin, bleomycin, streptonigrin, and albomycin are examples of such antibiotics. In some cases metal ions are attached to the antibiotic molecule without causing a major change in antibiotic structure. Tetracyclines, aureolic acids, and quinolones are frequently used in these strategies (Chohan et al., 2005). Synthetic or semi-synthetic antimicrobial compounds also possess metal ions in their structure.

In general, antimicrobials which contain metal compounds in their structure in a natural form (nature-occurring) or which have metal compounds incorporated synthetically are termed metalloantibiotics. Transition metals are generally preferred in metalloantibiotics and are present in very low concentration *in vivo*. The ligand environment of transition metal ions can generally change considerably upon administration of a therapeutically effective dose of an antibacterial drug (Sekhon, 2010). Some strategies are followed to synthesize metal nanoparticles using antibiotics as *in situ* reducing and capping agent. Here antibiotics act as an *in situ* reducing and capping agent, thus offering potent antimicrobial activities as well their application in antimicrobial coatings (Jagannathan et al., 2007; Rai et al., 2010). Although interactions with essential metal ions make more controllable conditions for bacterial infections, the unwanted side effects like toxicity and hemolytic activity sometimes increase simultaneously (Heidenau et al., 2005). So the potential for oral administration or internal use may be hampered.

## EFFLUX PUMP INHIBITORS

One of the most important strategies to combat bacterial resistance to antibiotics seems to be the efflux pump. Bacteria may pump the drug out of the cell after its entrance, and among the transporters involved in this pumping process are plasma membrane translocases. Being non-specific in nature such transporters are known as multi-drug-resistance pumps, being main determinants of the antibiotic concentration inside a bacterial cell. Many of them also act as drug/proton antiporters (protons enter the cell as the drug leaves). This is a very common resistance mechanism found in *Escherichia coli, P. aeruginosa, Mycobacterium smegmatis,* and *Staphylococcus aureus* (Takiff et al., 1996). Depending upon their varying structure and function, efflux pumps are subdivided into five classes: SMR pumps of the drug/metabolite transporters (DMTs) superfamily, ABC, RND, MFS, and MATE transporters of the multi-drug/oligosaccharidyl-lipid/polysaccharide flippases (MOP) superfamily (Piddock, 2006; Bhardwaj and Mohanty, 2012).

**FIGURE 5:**
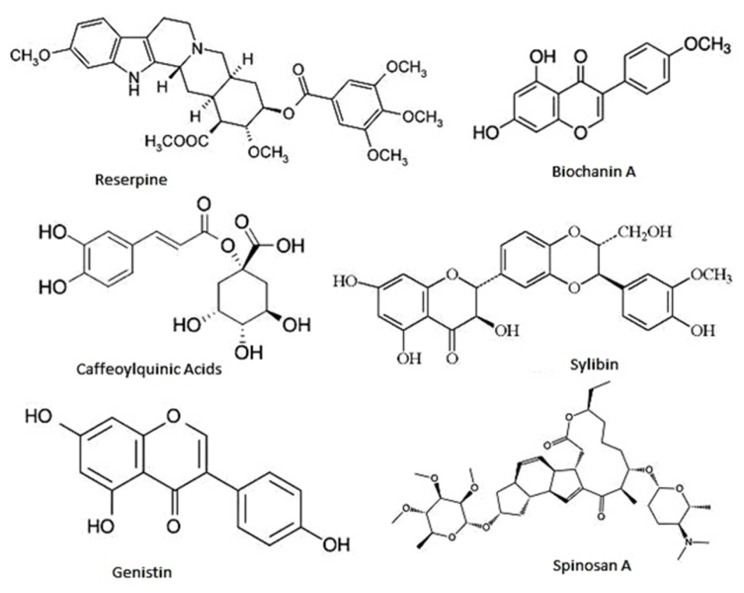
**Few representative chemical structures of efflux pump inhibitors**.

In order to restore the activity of antibiotics, an obvious strategy consists of developing a compound that inhibits the effects of efflux pump. Such molecules are named efflux pump inhibitors (**Figure 5**). In order to get effective results, several strategies have been taken including rational design of efflux pump inhibitors, their chemical synthesis and potentiality as combination with commercial antibiotics (Marquez, 2005; Delmar et al., 2014).

Although, there are few efflux pump inhibitors are available in market and research in progress raises the hope that they may be found in the near future. Screening of efflux pump inhibitors from natural origins or under synthetic production has attracted remarkable attention (Hudson et al., 2003). Structural modifications of such compounds may lead to an increase in the spectrum of activities A wide number of effective chemical compounds belonging to various chemical families have already showed efflux pump inhibition (Brincat et al., 2011; Holler et al., 2012a, b). Moreover, some plant-derived *NorA EPIs* and their chemical modifications are carried out effectively (Thota et al., 2008; Sabatini et al., 2011; Kalia et al., 2012). Recent rationally designed transmembrane peptide mimics may work in efflux pump inhibition. Maurya et al. (2013) have reported rationally designed transmembrane peptide mimics of the multi-drug transporter protein Cdr1. This acts as an antagonist to selectively block drug efflux and chemosensitize azole-resistant *Candida albicans* clinical isolates (Maurya et al., 2013). However, the major advantages of efflux pump inhibitors are that the possibilities on slower development of resistance by the target bacteria. Several disadvantages are also documented including their chemical synthesis due to bulky structure, solubility or permeability problems, required at higher concentration and chance of decreased the activity at one or both target sites for steric or electronic configurations, unless these molecules are carefully designed (Bremner, 2007).

## LPS INHIBITORS

The LPS layer or LPS in Gram-negative bacteria acts as a protective barrier. It prevents or slows down the entry of antibiotics and another toxic compound that could kill or injure bacteria (Miki and Hardt, 2013). LPS inhibitors are compounds that generally work by inhibiting 3 deoxy-D-manno-octulosonic acid 8-phosphate synthase (KDO 8-p synthase), an important enzyme in the LPS pathway. There have been several reports describing inhibitors, including PD404182 and polymyxin B (PMB), which are compounds that have been applied with antibiotic therapy, allowing the antibiotic to pass through the bacterial cell wall (Palmer and Rifkind, 1974; Lindemann, 1988; Birck et al., 2000).

Among the strategies for exploiting LPS inhibitors more fully, studies on bacterial enzymes that are essential for growth have recently provoked much interest and will certainly attract more attention in the near future in controlling bacterial resistance. Some recently discovered molecules like pedopeptins are promising LPS inhibitor candidates, preventing bacterial growth (Cheung et al., 2013; Kozuma et al., 2013). Some bacterial strains can control other LPS functions, such as the ability of various *Lactobacillus* strains to prevent *Salmonella* LPS-induced damage to the epithelial barrier function (Yeung et al., 2013). This drug is yet to be clinically validated with sufficient data. However, it is likely that this and other small molecules of animal or plant origin or synthetically engineered may be found and developed soon for use in inhibiting the translation machinery with an *in vitro* system.

## MYXOPYRONIN AND ARCHAEOCINS

Regulated gene expression is important in changing environments, stresses, and gene developmental programs. The activity of transcriptional factors enables RNA polymerases (RNAPs) to catalyze the transcription of DNA into RNA (Birck et al., 2000; Wiesler et al., 2012). Myxopyronin is an α-pyrone antibiotic produced by *Myxococcus fulvus* offering broad-spectrum antimicrobial activities for most Gram-positive species and some Gram-negative bacteria [*E. coli* D21f2tolC (rfa tolC), *Moraxella catarrhalis* ATCC25238]. It acts as inhibiting or binding bacterial RNAP by changing the structure of the RNAP switch region of the β-subunit of the enzyme. That renders the reading and transmitting DNA code inactive, resulting in bacterial control (Irschik et al., 1983; Campbell et al., 2001; Mukhopadhyay et al., 2008; Belogurov et al., 2009; Ho et al., 2009; Srivastava et al., 2011). Rifampin, an RNAP inhibitor in clinical utilization is capable of binds to the β-subunit of RNAP within the DNA/RNA channel and blocks the RNA elongation when the transcript converts two to three nucleotides in length (Campbell et al., 2001). It is a broad spectrum antimicrobial and is particularly active against *M. tuberculosis*. But, the major problem in treatment failure and fatal clinical outcome is due to the resistance to rifampin. The development of resistance to rifampin is due to mutations in 81 base pair (27 codons) of the β-subunit of RNAP (*rpoB*).

Archaea also contain potent antimicrobial compounds known as archaeocins, which are archaeal proteinaceous antimicrobials. Eight archaeocins from this family, among them halocins and sulfolobicins, have been partially or fully characterized showing antibacterial activity (O’Connor and Shand, 2002). The unique mode of action offered by these groups of antimicrobials draws remarkable attention as future antibiotic research. Besides, there are lot of unknown bacterial and archaeal sources yet to be explored. Thus discovery of new Myxopyronin and archaeocins hinges on recovery and cultivation of bacterial and archaeal organisms from the environment. Synthetic modification may lead this compound to become a future potent drug.

## CONCLUSION AND PROSPECTS

Growing concern about antibiotic resistance is propelling the urgent modification of existing antibiotics and parallel development of newer antibiotics. There are generally three inherent pipelines available to fight against antibiotic resistance; i.e., antimicrobial chemical weaponry from natural products, synthetic chemical compounds turned into antibiotics, and phages. Antimicrobial compounds from natural product (AMPs, phytochemicals, efflux pump inhibitors, LPS inhibitors myxopyronin, and Archaeocins) have drawbacks regarding their isolation and purification. The cost of production can be reduced by isolating potent compounds from natural origins and then synthesizing them, or by rationally modifying derived compounds. Phytochemicals isolated from natural sources and then chemically synthesized via modifications are likely to provide the most effective antimicrobial drugs in the near future.

New compounds that target bacterial virulence can be developed to control the enormous threat posed by multi-drug-resistance. Antibiotic structural modifications can be carried out by synthesizing potent structures from already existing antibiotics. Here the metalloantibiotics can play a great role.

In parallel, further research into toxicity against animal or human cells, mechanisms of action, *in vivo* effects, and negative and positive interactions with common antibiotics should be incorporated. The main challenge is to find the most effective techniques for isolating and purifying newer and safer naturally occurring antimicrobials against MDR pathogens. A better understanding of the structure, function and action mechanism of existing and newly identified AMPs will lead to their being fine-tuned by proper design to work against MDR pathogens. Phages may also play a major role in treating bacterial infections in humans. Combined treatment of phages with antibiotics is likely to be a future choice. The problem regarding expansion of phages can only be solved if large-scale clinical trials are carried out by major pharmaceutical companies.

In summary, it is imperative to develop new classes of antibiotic or antimicrobial agents with different modes of action against MDR pathogens. Combinational drug use is extensively used to treat bacterial infection, but even this combinational dose pattern may lead to resistance among pathogens. To overcome the challenges of antibiotic resistance, antimicrobial compounds with a new mechanistic approach should be urgently sought.

## Conflict of Interest Statement

The authors declare that the research was conducted in the absence of any commercial or financial relationships that could be construed as a potential conflict of interest.
